# The Life of Thomas Linacre, Doctor in Medicine, Physician to King Henry VIII

**Published:** 1835-07-01

**Authors:** 


*The Life of Thomas Linacre, Doctor in Medicine, Physician to King Henry VIII. The Tutor and Friend of Sir Thomas More, and the Founder of the College of Physicians in London. With memoirs of his Cotemporaries, and of the Rise and Progress of Learning; more particularly of the Schools, from the Ninth to the Sixteenth Century inclusive.* By JOHN NOBLE JOHNSON, M.D. Late Fellow of the Royal College of Physicians, London. Edited by Robert Graves, of the Inner Temple, Barrister-at-Law. London 1835. 8vo. pp. 363.

THE biography before us is a monument of the industry of its author, but contains very little which the most liberal construction can allow to find a place in a medical journal. We must, therefore, dismiss it with a very short notice; but we should be sorry to have passed it over without any; for the Founder of the College of Physicians was the link that connected the old and the modern practice of medicine. Like many, or most of his predecessors, he superadded the clerical profession to his own; but, by the institution of the College, he gave rank to the professors of physic, and "a system has consequently been constructed for the public service, which has now been carried on for three centuries, by which the character and respectability of physicians, and, through them, of the whole medical profession, has been raised to a higher eminence than in any other nation of Europe." (*Lives of British Physicians*, p. 10.)

The events of the life of Thomas Linacre may be summed up in a few lines. He was born, probably in 1460, at Canterbury, and went to Oxford in 1480, where four years afterwards he was elected a fellow of All Souls College. He then spent two years in Italy, and took the degree of M. D. at Padua. On his return he taught Greek in the University of Oxford; and, about the year 1501, King Henry VII. called him to court, as the tutor and physician of his son Prince Arthur. Our author comments upon this last fact in the following manner:

"These offices Linacre was invited to fill about the year 1501, and to them is said to have been added the still more important charge of the king's health in the capacity of domestic physician. As this trust not only constituted the highest honour, to which the members of the faculty of medicine could aspire, but involved in it the most important obligations, I shall offer a few remarks upon this presumed appointment, and upon the nature of the duty itself, as it existed in the middle of the fifteenth and early part of the sixteenth centuries.

"The unsettled state of physic as a science, before the revival of learning in the fifteenth century, rendered the practice of it rather a necessary accomplishment to the priesthood, with which it was generally united, than a distinct art cultivated on fixed and certain principles. To the ecclesiastics of the middle ages degrees in medicine conferred equal privileges with those in their proper faculty: but they gave to the possessor no claim to public confidence or to a remuneration for the services, which he might render by virtue of them; and the practice of the art was chiefly confined to men, who had seldom enjoyed the benefit of a scholastic education, or who boasted of acquirements in language, beyond a competent knowledge of the idiom and use of their vernacular tongue.

"The earliest mandate or warrant for the attendance of a physician at court, which the writer has been able to discover, is dated 33 Henry VI., a reign fertile in the patronage which was afforded to practitioners in medicine; but in that reign no appointment existed, which can justly be called physician to the royal person. By this warrant the king, with the consent of his privy council, deputed to three physicians, and two surgeons, the regulation of his diet and the administration of such medicines and remedies, as might be sufficient for his cure, without any allusion to the previous existence, or permanency of the office which they were authorized for a time to fill, or to a remuneration for their services. What was the nature of the malady, or what the reward of their efforts for its cure, does not appear. The king seems either to have been dissatisfied with the treatment which was adopted, or to have desired that spiritual consolation, in conjunction with medical advice, which could only be afforded by an ecclesiastic. In the following year, when he was seized either with a new disease, or an accession of his former complaint, he issued an order under his privy seal at Westminster, requiring the attendance of Gilbert Kemer, Dean of Salisbury, an expert, notable, and proved man in the craft of medicines, and in whom, amongst all others, the royal affection and desire is stated right specially to have been set." (P. 162.)

Dr. Johnson's style seems to us rather obscure; he means to say that medical practitioners at that time were divided into two classes, the clerical and the lay; of these the former had no legal claim to payment, and the latter were unlettered men.

The following note is very curious, on several accounts. It is pleasant to be doctored by a king, when his remedies are sovereign, angelic, and noble, in the monetary as well as the common sense of the words.

"In the Cotton library is a volume of extracts from an original book of accounts of Henry VIII., with the sign manual at the end of every month, supposed to be the accounts of Sir Orlando Bridgman, Lord Keeper, in the twentieth year of that reign. The following items from this MS., relating to the medical disbursements, confirm what has been said on this subject, and furnish some curious particulars of the state of medicine in this reign. His majesty himself seems to have been actively employed in the good work of healing; and it must be confessed that whatever honour accrued to the king's physicians, the principal share of the profit appears to have fallen to the lot of the apothecary, whose office, as the name implies, was then to provide medicines, without taking any share in the prescription of them.

21 Hen. 8, May 16, to Cuthbert, the king's apothecary in full, xxx. *l.* xii. *s.* vi. *d.*

Oct. 1, to Dr. Baugh, for two sick men at Waltham, xv. *shil.*

13, to the serjeant apothecary his bill, xxviii. *li.* iii. *s.* x. *d.*

23 Hen. 8, April 9, to Cuthberd, the king's apothecary, on his bill, xxx. *li.* iii. *s.* x. *d.*

July 28, p$^{d}$ to a poor child, the which the king's grace heled at Windsor, vii. *s.* vi. *d.*

Sept. 11, to two pooer folks that were heled of their sikeness, xv. *sh.*

18, to two pooer folke that the king's grace heled, xv. *sh.*

Oct. 23, to a pooer woman that the king's grace heled at Havering, vii. *sh.* vi. *d.*

— Hen. 8, Feb. 1, in reward to Dr. Yakesby and another physitian, iiii. *li.*

2, to my Lord Wiltshire, for a physitian called Dr. Nicholas, xx. *angells*, vii. *li.* x. *sh.*

March 30, p$^{d}$ to my lady princess physitian in reward, xxvi. *li.* xiii. *sh.*

— Hen. 8, April 3, to Cutberde, y$^{e}$ king's apothecary, on his bill, for stuff by him delivered for y$^{e}$ king's grace, from last Sept. to last Marche, xxxviii. *li.* vi. *s.* viii. *d.*

June 27, to a pooer woman that y$^{e}$ king heled of her sickness, vii. *sh.* vi. *d.*

July 21, to a pour child that the king heled of his sickness, vii. *sh.* vi. *d.*

Sept. 9, to the k$^{gs}$ apothecary for such stuff as he delivered to the k$^{gs}$ use, xxv. *li.* iiij. *sh.* vi. *d.*

Oct. 5, to Dr. Butts, physitian, for y^e use of Dr. Thurleby, B^p of Ely, by king's command, x. *li.*

"The king had studied the art of pharmacy with success, and in a MS. collection of recipes, principally for plasters and unguents, compiled by his physicians for the royal use (Sloane MSS. in Mus. Brit. No. 1047,) are several of his own invention. The intentions, which these remedies were to fulfil, are in unison with the hypotheses and pathology of the day, and the pharmaceutical efforts of the monarch were evidently directed towards an alleviation of the evils and infirmities of which an uncontrouled and continued indulgence of his passions had tended in the latter part of his life to render him the victim." (*Note*, p. 166.)

The sixth chapter of this book contains a very copious and interesting account of the foundation of the College of Physicians. A long note, of which we can afford to extract only a part, gives two cases of *plucking* in the sixteenth century, which may not be altogether void of instruction in the nineteenth; for they show how useful a second examination may prove as a test of the soundness of the first one.

"The history of this transaction, which is found in the annals of the college for the year 1555, affords a proof of the anxiety of its members to fulfil the intentions of the founder and to discharge the obligations to which they had bound themselves at their admission. The university of Oxford had admitted Simon Ludford, originally a Franciscan friar and afterwards an apothecary in London, and David Laughton, a coppersmith, two ignorant, unlettered, and incompetent persons, to the honours of the baccalaureate in medicine. The college reproved the university by letter, recommending that the vote which conferred the degrees should be rescinded, and advising a more cautious conduct in the future dispensation of them. With the former the university did not think it fit to comply, and the college was meditating further proceedings, when the inquisition of the Cardinal Pole, in 1556, for the reformation of religion and faith, and the correction of collegiate abuses, enabled them to prosecute their appeal with more effect. The college immediately laid their complaints before the visitors, to whom they gave the following specimen of Laughton's pretensions. 'Cujus infantia cum suggessit, ut quomodo corpus declinaretur, exigeremus, respondit —hic, hæc et hoc corpus, accusativo corporem;' adding, 'egregius certè ex universitate medicus, cui humana vita committeretur.'" (*Note*, p. 292.)

Linacre died in 1524, at the age of sixty-four, of ulceration of the bladder, and was buried in St. Paul's Cathedral.

Those who desire merely a brief abstract of the principal events in the life of Linacre, and of the services which he rendered to the republic of letters, and to the medical profession in particular, may be satisfied with the excellent biography contained in the Lives of British Physicians; but those who wish, in addition, for information respecting the physicians and scholars who flourished during the reigns of Henry VII. and VIII., should consult Dr. Johnson's work.

We think that the Editor has scarcely used sufficient diligence in correcting the Latin, which is frequently quoted: what, for instance, is the meaning of the latter half of the following sentence?

"Itaque te pro tuâ erga me benevolentiâ, et perpetuâ in omnes homines singulari humanitate peto et rogo, ut illum Titi Livii decades videre permittas; et ut perficias, quàm tum intelligat, has meas literas sibi si fortè voluit in emptione dicti operis multùm profuisse." (P. 7, *note.*)

